# 20th National Voice Campaign^☆^

**DOI:** 10.1016/j.bjorl.2018.09.001

**Published:** 2018-09-07

**Authors:** Gustavo Polacow Korn, Marcos André de Sarvat

**Affiliations:** a Universidade Federal de São Paulo (UNIFESP), São Paulo, SP, Brazil; b Academia Brasileira de Laringologia e Voz (ABLV), São Paulo, SP, Brazil; c Universidade Federal do Rio de Janeiro (UFRJ), Rio de Janeiro, RJ, Brazil; d Universidade Federal do Estado do Rio de Janeiro (UNIRIO), Rio de Janeiro, RJ, Brazil

On April 16, 2018, Brazil celebrated the 20th Edition of the Voice Campaign. Started in 1999, since 2003 it has gained international recognition in the form of the World Voice Day and, currently, more than 100 countries have adopted the Brazilian date. Created by Prof. Nédio Steffen, the National Voice Campaign/Week is the largest social event of Brazilian Otorhinolaryngology.

In the first two editions, the Campaign counted on the joint effort of doctors, speech therapists and singing professionals. This important partnership has decreased over the years and, surely, we all realized the need to resume it once again. Dozens of Services nationwide voluntarily participated in some editions, offering free care to the citizens during Voice Week, focusing on the early diagnosis of laryngeal cancer and detection of alterations, and speeding up the therapeutic approach. It should be noted that Brazil still exhibits a very high incidence of this type of neoplasm. The estimate for this year is of 7670 new cases.^1^

Throughout these 20 years, we have remained focused on the greater goal of the Voice Campaign, which utilizes guidance and awareness-raising activities to promote a lasting awareness of voice care. Each year means another brick in the construction and creation of this alert message – prevention and therapeutics. We must not fail to emphasize and register (and once again say “thank you”!) the free collaboration of prominent national public figures such as Xuxa, Sylvia Massari, Gabriel Pensador, Toni Garrido, Fernanda Abreu and Patricia Pillar (1999), Leila Pinheiro, Marília Pera, Thalma de Freitas, Flavia Monteiro, Tande, Parreira and Bernard (2001), Pelé and Assíria (2003), Lima Duarte (2004), Edson Celulari and Claudia Raia (2005), Andrea Beltrão (2006), Tarcisio Meira and Glória Menezes (2007), Fagner (2008 and 2009), Claudia Leite (2010), Daniela Mercury (2011), Deborah Secco (2012), Sandy Leah (2013), Fernando and Sorocaba (2014), Doutores da Alegria (2015), Wendel Bezerra (2016) and the Voice Club (2017). It was because of you, your credibility and generosity that we, all of us and our associations, have been able to guide and serve an increasingly higher number of citizens, earlier, and more effectively.

In 2010, an inflatable structure was developed, the giant larynx, in reality a giant mouth–pharynx–larynx model, that visitors can enter and learn about the anatomy of the throat and how the voice is produced, in an assemblage that represents an important brand of our Campaign. The structure has been modified twice and during these years has been, the highlight of the Campaign. Over the years, thousands of people, including children, have visited our inflatable model (Figure 1, Figure 2). And, in 2018, when we celebrated the 20th Campaign, we achieved a special highpoint, with the participation of our volunteers Ney Matogrosso, Elisa Lucinda, Maya Gabeira and Pedro Scooby, Geraldo Azevedo, Marisa Orth and Marcio Seixas in memorable TV and social media messages.

**Figure 1:**
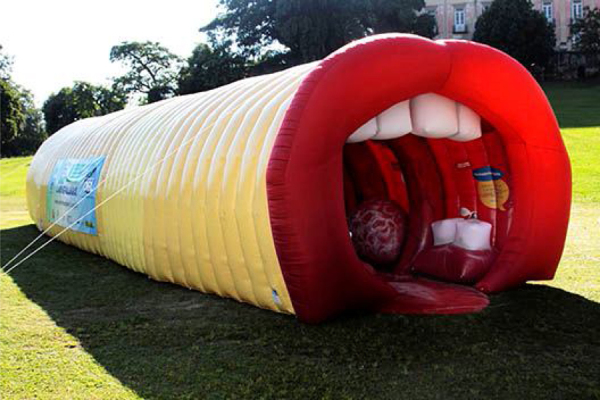
External view of the inflatable giant larynx.

**Figure 2:**
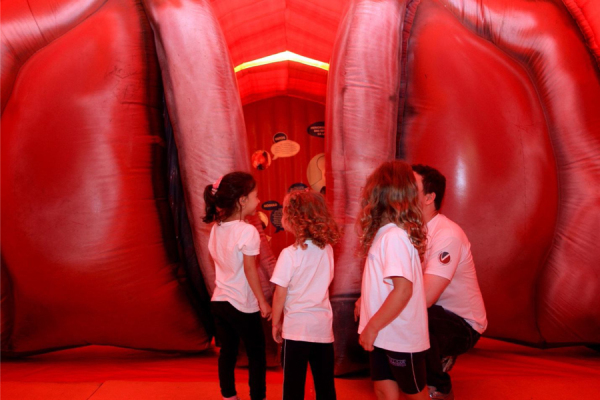
Inside view of the inflatable giant larynx, where the children observe the vocal folds.

The year 2018 has been an outstanding one for many reasons. Almost two hundred registered centers, including public hospitals and private clinics, assisted individuals between April 16 and 20; more than 3000 care forms were sent, and we participated in a beautiful opening ceremony at *Museu do Amanhã* in Rio de Janeiro. A historical association was developed among the ABORL-CCF (The Brazilian Association of Otorhinolaryngology and Cervical-Facial Surgery), the ABLV (Brazilian Academy of Larynx and Voice), the SBCCP (Brazilian Society of Head and Neck Surgery), INCA – Ministry of Health, Mouth and Throat Cancer Association (ACBG); the Brazilian Association of Singing (ABC), the Otorhinolaryngology Foundation (FORL) and the Institute of Otorhinolaryngology and Head and Neck Surgery (IOCP). The principal focus this year was to offer awareness and care to the population for the evaluation of the mouth, pharynx and larynx (vocal tract), free of charge, and to offer follow up, and, when necessary, referral for specialized treatment. In short, we emphasize that 2018 represented a record-breaking financial investment and we managed to reach many millions of citizens of this continental country with our guidelines on vocal hygiene and early diagnosis of diseases. Currently, many more people know the importance of knowing the causes of a sore throat, throat clearing, difficulty swallowing and hoarseness!

Both of us worked in the national coordination and were able to count on the commitment of state coordinators, whose collaboration was essential to make the entire 2018 Campaign a remarkable event, and to which we especially thank Dr. Hugo Lisboa Ramos, ABLV Vice-president, and the dedicated staff of ABORL-CCF, especially Mr. Renato Batista Sebastião, the manager of more than 10 editions of the National Voice Campaign.

We would like to thank you for the opportunity and confidence placed on us to coordinate the National Voice Campaign! It was a great honor and pleasure and an announcement can be made here: everyone, get ready for even better campaigns that highlight the importance of the specialty in all its fields! Tune up your health! Take care of your voice!

## Conflicts of interest

The authors declare no conflicts of interest.
